# Assessing risk factors for drug storage practices in veterinary surgeries: A questionnaire study of UK veterinary professionals

**DOI:** 10.1002/vro2.70005

**Published:** 2025-01-31

**Authors:** Jordan Perry, Kelly Gouveia

**Affiliations:** ^1^ School of Natural Sciences Faculty of Science, Business and Enterprise University of Chester Chester Cheshire UK

## Abstract

**Background:**

Safe drug storage practices are essential in veterinary practice to maintain optimal standards of animal care. Practical challenges in clinic may impact their uptake, which could affect drug efficacy and the success of treatment. The UK is presumed to provide high standards for animal care and welfare in the veterinary profession and may provide an interesting case study to assess veterinary drug storage practices.

**Methods:**

An online survey with 184 practice participants assessed UK veterinary professionals’ responses on drug storage practices. This included socio‐demographic information and questions or statements that examined storage practices compliant with the requirements established by the Royal College of Veterinary Surgeons (RCVS), the regulatory body for veterinary practice in the UK.

**Results:**

Overall, practices followed RCVS‐recommended measures, though there was clear selectivity for stock temperature checks (72.2%), over other measures, particularly, replacing stock (54.4%) and returning medications to refrigerated storage (52.5%). Clinical experience and practice type impacted most on drug storage practices, with more experienced clinicians and small animal practices showing greater uptake of best measures.

**Conclusions:**

We suggest that practices should adopt all of the RCVS measures in relation to veterinary drug storage practices. Socio‐demographic factors should be considered because they can act as risk factors influencing best practice in clinics. Our findings may have wider implications for the veterinary profession in general, given similar demographic trends in veterinary practice in many European countries.

## INTRODUCTION

In clinical veterinary practice, risk factors for storage practices have been mostly associated with human error,^1^, ^2^ requiring veterinary professionals to undergo appropriate undergraduate training, while following best principles on effective drug storage. The UK presents an interesting case study, because of the general expectations of high animal care and welfare standards it holds as a nation, and because historically it is known as one of the first countries to have legislated the veterinary profession. Therefore, assessing the current status of drug storage practices among UK veterinary professionals may provide a valuable benchmark for assessing potential strengths and limitations in the veterinary profession.

In the UK, the use and storage of veterinary medicines are regulated by the Veterinary Medicines Regulation (2013) (S.I. 2013/2033)^3^ and are enforced by the Veterinary Medicines Directorate. The regulatory body for the veterinary profession in the UK, the Royal College of Veterinary Surgeons (RCVS), assists with the enforcement of drug storage practices by establishing the legal requirements and standards of professional practice that professionals must follow to be members of the RCVS.^4^, ^5^, ^6^ They also provide guidance on best drug storage practices for veterinary professionals.^7^

There is limited information on how best practices are up‐taken in veterinary clinics.^8^ It is important to understand the extent to which such storage practices are up‐taken and identify potential risk factors that could potentially compromise best practice. Most studies on veterinary drug storage practices have focused on the impacts of drug use on‐farm and in particular associated with practices of the farmers.^9^, ^10^, ^11^ There is a need to consider storage practices across the wider veterinary profession.

Socio‐demographic factors could influence a veterinary professional's knowledge and attitude toward drug safety measures. Professional experience may play a role in determining attitudes towards storage practices, for example, newly qualified individuals may be less confident^12^, ^13^ or less aware of effective storage practices. The most experienced professionals are likely to be in managerial roles in veterinary practice and will be more invested in ensuring compliance with best practice. Drug storage practices may differ among different types of veterinary practices. For instance, the widespread use of cooling boxes in large animal practice to transport medications between livestock farms has the potential to compromise drug efficiency if the temperature within the box or vehicle exceeds the drug's stability.^1^ There could be gender differences in storage practices and performance of best practice, given that the veterinary profession is largely female dominated.^14^, ^15^

We aimed to investigate the uptake of best drug storage practices, in accordance with RCVS guidance, within UK veterinary surgeries. Individual veterinary practices were recruited to participate in an online survey. The study examined the impact of socio‐demographic characteristics, including the level of professional experience, veterinary practice type, professionals’ age, gender or profession (veterinary surgeon or nurse) on drug storage practices. We hypothesised that mostly RCVS standards were likely to be adhered due to this being a legal requirement; further that more experienced professionals, in particular practice managers, would potentially have better storage practices due to their legal responsibility for the veterinary practice, also given their greater knowledge and experience.

## METHODS

### Data collection

An online questionnaire was created to assess drug storage practices and effect of socio‐demographic factors on storage practices. Veterinary practices in the UK were contacted (*n* = 250), including small, large, mixed and exotic types. Veterinary professionals, veterinary surgeons and nurses, were recruited because both professions are legally responsible for safe drug storage practices. All practices were registered with the RCVS and were contacted via email, through a contact list provided by the RCVS. In total, 189 questionnaires were collated, accounting for an uptake of 76% of questionnaires distributed from November 2020 until March 2021. To ensure maximum uptake of the survey, we sent out an email reminder to participants 2 weeks after the initial invitation. This study was approved by the Department of Biological Sciences at the University of Chester, research ethics committee. Participation was voluntary and informed consent was included in the introductory letter of the questionnaires, which also ensured participant confidentiality at all stages of the study.

### Questionnaire

The questionnaire comprised 14 closed end questions with a single option to choose from and was divided into two sections. The first section consisted of the socio‐demographic characteristic of the participant, including age, gender, years of professional experience, profession (veterinary surgeon or nurse). We included socio‐demographic factors, such as practice type (small, large animal, exotic and mixed). We investigated the role of dispenser nurse because this role in human medicine has shown to affect the efficiency of drug storage and administration.^16^ For this, we first determined the uptake of this designated role in veterinary practice; second we assessed whether there was an association between this socio‐demographic factor and best practices in veterinary drug storage.

The second section examined the drug storage practices of veterinary professionals against best practices as advised by the RCVS.^7^ A Likert scale was used for responses, which allowed participants to choose an answer that best represented their practices and knowledge on best practice for storing veterinary medicines (see Supporting Information S1).

### Statistical analysis

Data were analysed using IBM SPSS statistics version 22.0 software package. Univariate descriptive analysis examined the uptake of RCVS standards. Chi‐square association tests assessed the relationship between the participant responses and socio‐demographic factors. The ‘prefer not to say’ survey responses were treated as missing and were excluded from the analysis. As there was only one exotic practice, this was eliminated from the statistical analysis assessing associations between practice type and storage practices. *p*‐Values were calculated for differences in responses between the four age groups, clinical experience groups, gender, practice type and profession, for each standard.

## RESULTS

### Demographic information of participants

Most participants were veterinary surgeons (*n* = 138, 78.0%) and just under one third were veterinary nurses (*n* = 39, 22.0%). Most respondents were female (*n* = 109, 61.0%) and just over one third were male (*n* = 70, 39.1%). Participant distribution among age categories was similar, with approximately one third of participants in each age category (Table S1). The most common type of clinical practice was small animal (*n* = 121, 67.6%), followed by large animal (*n* = 33, 18.4%), mixed (*n* = 24, 13.4%) and lastly exotics (*n* = 1, 0.6%). Participants varied in their clinical experience, with most having over 20 years of experience (*n* = 71, 39.7%) and around one quarter had between 10 and 19 years (*n* = 41; 22.9%), fewer participants were least experienced (5–9 years, *n* = 32, 17.9%; <5 years, *n* = 34, 19.0%). There were dispensary nurses in some practices (*n* = 33, 18.2%).

### Participants responses to veterinary drug storage practices

The responses collected suggested that veterinary professionals were generally following legislative requirements on storage practices within most practices. For instance, 81.1% of participants (*n* = 146) reported always adhering to the veterinary medicine's storage requirements; 72.2% (*n* = 130) regularly checked maximum and minimum temperature for storage equipment; 68.7% (*n* = 123) always recorded the veterinary medicine's delivery date, usage in practice and expiration date (Table 1). In other respects, practices were less adherent, with 40.0% (*n* = 70) recording expenditure wastage, 52.5% (*n* = 94) returning a veterinary medicine to the correct storage immediately after use and 32.2.% (*n* = 54) storing veterinary medicines at least sometimes in a vehicle overnight (Table 1).

**TABLE 1:** Participant percentages for each storage practice.

	Percentage of respondents					
Drug practice question (dependant variable)	Total *n* (%)	Never	Rarely	Sometimes	Often	Always
‘All medications are returned to the correct storage immediately after use’	179 (100)	0 (0)	4 (2.2)	8 (4.5)	73 (40.8)	94 (52.5)
‘Advised storage requirements (e.g., cool temperature 8°C–15°C) for all medications are adhered to’	180 (100)	0 (0)	1 (0.6)	2 (1.1)	31 (17.2)	146 (81.1)
Is drug storage checked for current maximum and minimum temperatures?	180 (100)	1 (0.6)	2 (1.1)	12 (6.7)	35 (19.4)	130 (72.2)
‘Medications are replaced when the manufacturers storage requirements are exceeded’	180 (100)	1 (0.6)	5 (2.8)	24 (13.3)	52 (28.9)	98 (54.4)
Do you record expenditure wastage in case a veterinary medicine is spoiled?	175 (100)	16 (9.1)	19 (10.9)	31 (17.7)	39 (22.3)	70 (40.0)
When stock arrives, is the expiry date checked?	179 (100)	0 (0)	8 (4.5)	16 (8.9)	42 (23.5)	113 (63.1)
Is there a system in place to record drug delivery, expiration dates and usage within practice (i.e., computer database, paper documentation)?	179 (100)	11 (6.1)	2 (1.1)	19 (10.6)	24 (13.4)	123 (68.7)
Are drugs stored within a vehicle overnight or for a period of time?	180 (100)	117 (65.0)	5 (2.8)	9 (5.0)	24 (13.3)	25 (13.9)

### Associations between clinical experience, profession and storage practices

There was an overall statistical association between clinical experience and participant responses to drug storage practices. Regardless of profession, the most experienced professionals (over 20 years) reported always storing medications immediately following use, while those who were less experienced (with 5, 6–9, 10–19 years) reported more variable responses to this practice (*p* = 0.04; Table 2). Experienced professionals were more consistent with replacing medications when manufacturers guidance was exceeded (*p *< 0.001); also checking the maximum and minimum temperatures of surgery refrigerators (*p* = 0.014) and ensuring correct temperatures were adhered to in storage (*p* < 0.001) in comparison to less experienced professionals. Profession in veterinary practice did not impact significantly on the uptake of any drug storage practice (Table 3).

**TABLE 2:** Associations between clinical experience and drug storage practices.

Storage practices	Total *n* (%)	Never	Rarely	Sometimes	Often	Always	*p*‐Value
Less than 5 years							
Storage after use	34 (19)	0 (0)	2 (1.1)	1 (0.6)	22 (12.3)	9 (5.0)	
Temperature storage	34 (18.9)	0 (0)	0 (0)	2 (1.1)	14 (7.8)	18 (10.0)	
Temperature check	34 (18.9)	1 (0.6)	1 (0.6)	4 (2.2)	8 (4.4)	20 (11.1)	
Replaced medicines	34 (18.9)	0 (0)	2 (1.1)	5 (2.8)	12 (6.7)	15 (8.3)	
Recording expenditure wastage	33 (18.9)	4 (2.3)	3 (1.7)	6 (3.4)	7 (4.0)	13 (7.4)	
Expiry date check	34 (19.0)	0 (0)	3 (1.7)	5 (2.8)	3 (1.7)	23 (12.8)	
Recording expiry date	34 (19.0)	4 (2.2)	1 (0.6)	4 (2.2)	4 (2.2)	21 (11.7)	
Vehicle storage	34 (18.9)	21 (11.7)	1 (0.6)	0 (0)	6 (3.3)	6 (3.3)	

5–9 years						
Storage after use	32 (17.9)	0 (0)	0 (0)	1 (0.6)	17 (9.5)	14 (7.8)
Temperature storage	32 (17.8)	0 (0)	0 (0)	0 (0)	9 (5.0)	23 (12.8)
Temperature check	32 (17.8)	0 (0)	1 (0.6)	3 (1.7)	5 (2.8)	23 (12.8)
Replaced medicines	32 (17.8)	1 (0.6)	1 (0.6)	4 (2.2)	12 (6.7)	14 (7.8)
Recording expenditure wastage	31 (17.7)	4 (2.3)	3 (1.7)	6 (3.4)	8 (4.6)	10 (5.7)
Expiry date check	32 (17.9)	0 (0)	1 (0.6)	1 (0.6)	7 (3.9)	23 (12.8)
Recording expiry date	31 (17.3)	1 (0.6)	0 (0)	4 (2.2)	3 (1.7)	23 (12.8)
Vehicle storage	32 (17.8)	19 (10.6)	1 (0.6)	0 (0)	8 (4.4)	4 (2.2)

10–19 years						
Storage after use	41 (22.9)	0 (0)	1 (0.6)	3 (1.7)	14 (7.8)	23 (12.8)
Temperature storage	41 (22.8)	0 (0)	1 (0.6)	0 (0)	4 (2.2)	36 (20.0)
Temperature check	41 (22.8)	0 (0)	0 (0)	1 (0.6)	12 (6.7)	28 (15.6)
Replaced medicines	41 (22.8)	0 (0)	0 (0)	7 (3.9)	9 (5.0)	25 (13.9)
Recording expenditure wastage	41 (23.4)	5 (2.9)	6 (3.4)	4 (2.3)	10 (5.7)	16 (9.1)
Expiry date check	41 (22.9)	0 (0)	3 (1.7)	4 (2.2)	13 (7.3)	21 (11.7)
Recording expiry date	40 (22.3)	1 (0.6)	0 (0)	6 (3.4)	7 (3.9)	26 (14.5)
Vehicle storage	41 (22.8)	25 (13.9)	1 (0.6)	5 (2.8)	4 (2.2)	6 (3.3)

20+ years							
Storage after use	71 (39.7)	0 (0)	1 (0.6)	3 (1.7)	20 (11.2)	47 (26.3)	0.042
Temperature storage	72 (40.0)	0 (0)	0 (0)	0 (0)	4 (2.2)	68 (37.8)	<0.001
Temperature check	72 (40.0)	0 (0)	0 (0)	3 (1.7)	10 (5.6)	59 (32.8)	0.014
Replaced medicines	72 (40.0)	0 (0)	1 (0.6)	8 (4.4)	19 (10.6)	44 (24.4)	<0.001
Recording expenditure wastage	69 (39.4)	2 (1.1)	7 (4.0)	15 (8.6)	14 (8.0)	31 (17.7)	0.310
Expiry date check	71 (39.7)	0 (0)	1 (0.6)	6 (3.4)	18 (10.1)	46 (25.7)	0.216
Recording expiry date	73 (40.8)	4 (2.2)	1 (0.6)	5 (2.8)	10 (5.6)	53 (29.6)	0.098
Vehicle storage	72 (40.0)	51 (28.3)	2 (1.1)	4 (2.2)	6 (3.3)	9 (5.0)	

*Note*: Most experienced professionals with over 20 years of experience in veterinary clinics showed better drug storage practices in comparison to less experienced professionals. *p*‐Values show comparisons between all clinical experience groups for each storage practice.

**TABLE 3:** Associations between profession and storage practices.

Storage practices	Total *n* (%)	Veterinarian					Total *n* (%)	Veterinary nurse					
		Never	Rarely	Sometimes	Often	Always		Never	Rarely	Sometimes	Often	Always	*p*‐value
Storage after use	138 (78.0)	0 (0)	2 (1.1)	8 (4.5)	56 (31.6)	72 (40.7)	39 (22)	0 (0)	2 (1.1)	0 (0)	17 (9.6)	20 (11.3)	0.245
Temperature storage	139 (78.1)	0 (0)	1 (0.6)	1 (0.6)	27 (15.2)	110 (61.8)	39 (21.9)	0 (0)	0 (0)	1 (0.6)	4 (2.2)	34 (19.1)	0.405
Temperature check	139 (78.1)	1 (0.6)	1 (0.6)	11 (6.2)	27 (15.2)	99 (55.6)	39 (21.9)	0 (0)	1 (0.6)	1 (0.6)	7 (3.9)	30 (16.9)	0.615
Replaced medicines	139 (78.1)	1 (0.6)	4 (2.2)	20 (11.2)	41 (23)	73 (41.0)	39 (21.9)	0 (0)	1 (0.6)	4 (2.2)	10 (5.6)	24 (13.5)	0.863
Recording expenditure wastage	134 (77.5)	15 (8.7)	16 (9.2)	20 (11.6)	29 (16.8)	54 (31.2)	39 (22.5)	1 (0.6)	3 (1.7)	10 (5.8)	9 (5.2)	16 (9.2)	0.291
Expiry date check	138 (78.0)	0 (0)	6 (3.4)	11 (6.2)	29 (16.4)	92 (52.0)	39 (22.0)	0 (0)	2 (1.1)	5 (2.8)	12 (6.8)	20 (11.3)	0.359
Recording expiry date	138 (78.0)	10 (5.6)	2 (1.1)	12 (6.8)	18 (10.2)	96 (54.2)	39 (22.0)	1 (0.6)	0 (0.0)	7 (4.0)	5 (2.8)	26 (14.7)	0.392
Vehicle storage	139 (78.1)	83 (46.6)	4 (2.2)	6 (3.4)	22 (12.4)	24 (13.5)	39 (21.9)	32 (18.0)	1 (0.6)	3 (1.7)	2 (1.1)	1 (0.6)	0.035

*Note*: Profession did not impact on drug storage practices in veterinary surgeries. *p*‐Values show comparisons between professions for each storage practice.

### Associations between age, gender and storage practices

We found a statistical association between participant age and responses to some storage practices in veterinary practices (Table 4). Older participants had a higher percentage reporting always storing veterinary medications directly after use in comparison to younger professionals (*p* = 0.04). Respondents within the 50+‐year group had the highest percentage for always storing medications after use (73% within age category; Table 4), while younger age groups were less willing to follow this practice, as shown by 55.6% of the 40–49 age group, 45.8% of the 30–39 age group and 34.1% of the 20–29 age group.

**TABLE 4:** Associations between age and storage practices.

20–29 years							
Storage practices	Total *n* (%)	Never	Rarely	Sometimes	Often	Always	*p*‐Value
Storage after use	41 (22.9)	0 (0)	2 (1.1)	1 (0.6)	24 (13.4)	14 (7.8)	
Temperature storage	41 (22.8)	0 (0)	0 (0)	2 (1.1)	12 (6.7)	27 (15.0)	
Temperature check	41 (22.8)	1 (0.6)	2 (1.1)	4 (2.2)	7 (3.9)	27 (15.0)	
Replaced medicines	41 (22.8)	0 (0)	3 (1.7)	4 (2.2)	13 (7.2)	21 (11.7)	
Recording expenditure wastage	41 (23.4)	5 (2.9)	2 (1.1)	10 (5.7)	9 (5.1)	15 (8.6)	
Expiry date check	41 (22.9)	0 (0)	3 (1.7)	5 (2.8)	7 (3.9)	26 (14.5)	
Recording expiry date	41 (22.9)	5 (2.8)	1 (0.6)	5 (2.8)	5 (2.8)	25 (14.0)	
Vehicle storage	41 (22.8)	29 (16.1)	1 (0.6)	0 (0)	5 (2.8)	6 (3.3)	

30–39 years						
Storage after use	48 (26.8)	0 (0)	1 (0.6)	3 (1.7)	22 (12.3)	22 (12.3)
Temperature storage	48 (26.7)	0 (0)	1 (0.6)	0 (0)	15 (8.3)	32 (17.8)
Temperature check	48 (26.7)	0 (0)	0 (0)	4 (2.2)	13 (7.2)	31 (17.2)
Replaced medicines	48 (26.7)	1 (0.6)	0 (0)	11 (6.1)	14 (7.8)	22 (12.2)
Recording expenditure wastage	46 (26.3)	4 (2.3)	8 (4.6)	5 (2.9)	11 (6.3)	18 (10.3)
Expiry date check	48 (26.8)	0 (0)	1 (0.6)	4 (2.2)	12 (6.7)	31 (17.3)
Recording expiry date	47 (26.3)	1 (0.6)	0 (0)	7 (3.9)	6 (3.5)	33 (18.4)
Vehicle storage	48 (26.7)	26 (14.4)	2 (1.1)	2 (1.1)	10 (5.6)	8 (4.4)

40–49 years						
Storage after use	45 (25.1)	0 (0)	1 (0.6)	3 (1.7)	16 (8.9)	25 (14.0)
Temperature storage	45 (25.0)	0 (0)	0 (0)	0 (0)	4 (2.2)	41 (22.8)
Temperature check	45 (25.0)	0 (0)	0 (0)	1 (0.6)	7 (3.9)	37 (20.6)
Replaced medicines	45 (25.0)	0 (0)	0 (0)	5 (2.8)	11 (6.1)	29 (25.0)
Recording expenditure wastage	44 (25.1)	4 (2.3)	4 (2.3)	6 (3.4)	11 (6.3)	19 (10.9)
Expiry date check	44 (24.6)	0 (0)	3 (1.7)	4 (2.2)	13 (7.3)	24 (13.4)
Recording expiry date	44 (24.6)	2 (1.1)	0 (0)	3 (1.7)	8 (4.5)	31 (17.3)
Vehicle storage	45 (25.0)	29 (16.1)	0 (0)	5 (2.8)	5 (2.8)	6 (3.3)

50+ years							
Storage after use	45 (25.1)	0 (0)	0 (0)	1 (0.6)	11 (6.1)	33 (18.4)	0.041
Temperature storage	46 (25.6)	0 (0)	0 (0)	0 (0)	46 (25.6)	46 (25.6)	<0.001
Temperature check	46 (25.6)	0 (0)	0 (0)	3 (1.7)	8 (4.4)	35 (19.4)	0.208
Replaced medicines	46 (25.6)	0 (0)	2 (1.1)	4 (2.2)	14 (7.8)	26 (14.4)	0.218
Recording expenditure wastage	44 (25.1)	3 (1.7)	5 (2.9)	10 (5.7)	8 (4.6)	18 (10.3)	0.781
Expiry date check	46 (25.7)	0 (0)	1 (0.6)	3 (1.7)	10 (5.6)	32 (17.9)	0.780
Recording expiry date	47 (26.3)	3 (1.7)	1 (0.6)	4 (2.2)	5 (2.8)	34 (19.0)	0.685
Vehicle storage	46 (25.6)	33 (18.3)	2 (1.1)	2 (1.1)	4 (2.2)	5 (2.8)	

*Note*: Older participants, over 50 years old, reported slightly better drug storage practices on certain measures, particularly storing medications immediately following use and ensuring correct storage temperatures were adhered to, in comparison to younger professionals who showed more variables responses to these drug storage practices. *p*‐Values show comparisons between all age groups for each storage practice.

Adhering to correct storage temperatures varied significantly across age groups (*p* < 0.001). With ensuring correct storage temperatures, older professionals (50+ years 100% and 40–49 years 91%) reported higher levels of adherence compared with younger professionals (30–39 years 66.7% and 20–29 years 65.9%). Replacing medications, recording expenditure wastage, checking maximum and minimum temperatures, and recording the expiry date of drugs were all adhered to equally by all age groups (Table 4). There was no overall effect of gender on participant responses to storage practices (Table 5).

**TABLE 5:** Associations between gender and storage practices.

		Gender											
Storage practices	Total *n* (%)	Male					Total *n* (%)	Female					*p*‐value
		Never	Rarely	Sometimes	Often	Always		Never	Rarely	Sometimes	Often	Always	
Storage after use	70 (39.1)	0 (0)	1 (0.6)	2 (1.1)	25 (14)	42 (23.5)	109 (60.9)	0 (0)	3 (1.7)	6 (3.4)	48 (26.8)	52 (29.1)	0.399
Temperature storage	70 (38.9)	0 (0)	0 (0)	0 (0)	8 (4.4)	62 (34.4)	110 (61.1)	0 (0)	1 (0.6)	2 (1.1)	23 (12.8)	84 (46.7)	0.177
Temperature check	70 (38.9)	1 (0.6)	0 (0)	3 (1.7)	9 (5.0)	57 (31.7)	110 (61.1)	0 (0)	2 (1.1)	9 (5.0)	26 (14.4)	73 (40.6)	0.102
Replaced medicines	70 (38.9)	1 (0.6)	3 (1.7)	5 (2.8)	20 (11.1)	41 (22.8)	110 (61.1)	0 (0)	2 (1.1)	19 (10.6)	32 (17.8)	57 (31.7)	0.187
Recording expenditure wastage	69 (39.4)	8 (4.6)	6 (3.4)	11 (6.3)	15 (8.6)	29 (16.6)	106 (60.6)	8 (4.6)	13 (7.4)	20 (11.4)	24 (13.7)	41 (23.4)	0.814
Expiry date check	70 (39.1)	0 (0)	3 (1.7)	6 (3.4)	18 (10.1)	43 (24.0)	109 (60.9)	0 (0)	5 (2.8)	10 (5.6)	24 (13.4)	70 (39.1)	0.955
Recording expiry date	69 (38.5)	4 (2.2)	1 (0.6)	4 (2.2)	8 (4.5)	52 (29.1)	110 (61.5)	7 (3.9)	1 (0.6)	15 (8.4)	16 (8.9)	71 (39.7)	0.465
Vehicle storage	70 (38.9)	47 (26.1)	2 (1.1)	3 (1.7)	6 (3.3)	12 (6.7)	110 (61.1)	70 (38.9)	3 (1.7)	6 (3.3)	18 (10.0)	13 (7.2)	0.554

*Note*: Gender of the professional did not influence the uptake of different drug storage practices in veterinary surgeries. *p*‐Values show comparisons among gender for each storage practice.

### Associations between practice type and storage practices

Small companion animal practices followed best storage practices more consistently, overall, than large and mixed practices (Table 6). This was particularly with respect to correct storage temperatures (*p* < 0.011; Table 6). Specifically, less professionals from mixed (77.8%) and large animal practices (60.6%) reported following this practice, in comparison to small animal practices (88.5%). Similarly, there was a difference among practices in replacing medications when manufacturers’ storage requirements were exceeded (*p* = 0.008), with small animal practices (63.1%) reporting higher levels in contrast to mixed (37.5%) and large animal practices (33.3%).

**TABLE 6:** Associations between practice type and storage practices.

Large animal practice							
Storage practices	Total *n* (%)	Never	Rarely	Sometimes	Often	Always	*p*‐Value
Storage after use	33 (18.5)	0 (0)	1 (0.6)	3 (1.7)	16 (9.0)	13 (7.3)	
Temperature storage	33 (18.4)	0 (0)	1 (0.6)	0 (0)	12 (6.7)	20 (11.2)	
Temperature check	33 (18.4)	0 (0)	0 (0)	4 (2.2)	8 (4.5)	21 (11.7)	
Replaced medicines	32 (18.4)	1 (0.6)	1 (0.6)	8 (4.5)	12 (6.7)	11 (6.1)	
Recording expenditure wastage	32 (18.4)	3 (1.7)	4 (2.3)	4 (2.3)	6 (3.4)	15 (8.6)	
Expiry date check	33 (18.5)	0 (0)	1 (0.6)	2 (1.1)	2 (1.1)	28 (15.7)	
Recording expiry date	31 (17.4)	1 (0.6)	0 (0)	1 (0.6)	5 (2.8)	24 (13.5)	
Vehicle storage	33 (18.4)	2 (1.1)	0 (0)	2 (1.1)	13 (7.3)	16 (8.9)	

Small animal practice						
Storage after use	121 (68.0)	0 (0)	3 (1.7)	3 (1.7)	45 (25.3)	70 (39.3)
Temperature storage	122 (68.2)	0 (0)	0 (0)	2 (1.1)	12 (6.7)	108 (60.3)
Temperature check	122 (68.2)	1 (0.6)	2 (1.1)	7 (3.9)	20 (11.2)	92 (51.4)
Replaced medicines	119 (68.4)	0 (0)	4 (2.2)	8 (4.5)	33 (18.4)	77 (43.0)
Recording expenditure wastage	119 (68.4)	12 (6.9)	11 (6.3)	22 (12.6)	27(15.5)	47 (27.0)
Expiry date check	121 (68.0)	0 (0)	6 (3.4)	10 (5.6)	35 (19.7)	70 (39.3)
Recording expiry date	123 (69.1)	8 (4.5)	1 (0.6)	15 (8.4)	17 (9.6)	82 (46.1)
Vehicle storage	122 (68.2)	112 (62.6)	5 (2.8)	3 (1.7)	2 (1.1)	0 (0)

Mixed practice							
Storage after use	24 (13.5)	0 (0)	0 (0)	2 (1.1)	12 (6.7)	10 (5.6)	0.259
Temperature storage	24 (13.4)	0 (0)	0 (0)	0 (0)	7 (3.9)	17 (9.5)	0.002
Temperature check	24 (13.4)	0 (0)	0 (0)	1 (0.6)	7 (3.9)	16 (8.9)	0.649
Replaced medicines	24 (13.4)	0 (0)	0 (0)	8 (4.5)	7 (3.9)	9 (5.0)	0.001
Recording expenditure wastage	23 (13.2)	1 (0.6)	4 (2.2)	4 (2.2)	6 (3.4)	8 (4.6)	0.911
Expiry date check	24 (13.5)	0 (0)	1 (0.6)	4 (2.2)	5 (2.8)	14 (7.9)	0.085
Recording expiry date	24 (13.5)	2 (1.1)	1 (0.6)	3 (1.7)	1 (0.6)	17 (9.6)	0.516
Vehicle storage	24 (13.4)	2 (1.1)	0 (0)	4 (2.2)	9 (5.0)	9 (5.0)	

*Note*: Small animal clinical professionals were more prompt with adhering to correct storage temperatures and replacing medications that exceeded manufacturers’ guidance in contrast to professionals working in mixed and large animal practice. *p*‐Values show comparisons between three practice types for each storage practice.

### Associations between dispenser nurse and storage practices

Having a designated dispenser nurse in veterinary practice did not make a difference on participants’ responses to drug storage practices (Table S2).

## DISCUSSION

Mostly participants prioritised drug storage practices associated with temperature checks over others, in particular returning medications to storage immediately following use, replacing medications when manufacturers’ requirements were exceeded or checking expiry dates. While performing temperature checks is indeed an essential safekeeping measure, as temperature abuses can easily contribute to the potential spoilage of veterinary medicines,^8^ it is equally important to carry out different measures collectively for best storage practice. Medications should be stored immediately especially those requiring refrigeration. Likewise, checking expiry dates of medications is important, from the time the medication arrives at the clinic until the medication is submitted for disposal.

Clinical experience had the most statistically significant impact on drug storage practices. Respondents with over 20 years of clinical experience most often reported following best practice as advised by the RCVS. This finding is in agreement with our hypothesis that clinical experience is likely to warrant better practices in general among veterinary professionals. It is consistent with studies in human medicine.^16^ However, more experienced professionals are likely to also be practice owners,^17^ they could have greater financial investment in ensuring best practice is undertaken to prevent financial burdens of drug spoilage and wastage, to safeguard the reputation of their business. Indeed, it has been reported that veterinary science courses and training are becoming orientated towards developing professional skills including communication, finances and business management.^18^ Ultimately, having a more business‐oriented approach to professional practice combined with extensive clinical experience and potential ownership of a veterinary practice could be vital factors in promoting best drug storage practices in veterinary science.

Overall, profession had little influence on the uptake of best drug storage practices in veterinary practices, which shows similar understanding and professional attitude towards drug safety measures among veterinary nurses and surgeons. Yet, both roles have different responsibilities and expectations in veterinary practice,^19^, ^20^ primarily enforced by their education, training and employment. One such difference is the length of study, which is greater in medical students (5 years) in comparison to nursing students (3–4 years). This could impact on the level of learning and understanding of general pharmacology principles, because the veterinary nursing course is heavily focused on the supportive care of animals and the administration of medications as directed by the veterinarian,^7^ whereas in a lengthier course, veterinary students have the opportunity for more comprehensive learning on drug pharmacology.^21^ Yet, in small animal practice veterinary nurses are usually the first‐line consultants in vaccination consultations, possibly explaining why they were as prompt to check temperature of refrigerators in consultation rooms and in the drug storage area as veterinary surgeons. Our study suggests that despite differences in educational background among professions, overall professional understanding and attitudes towards drug storage practices are just as well‐established in both professions.

Small animal practices seemed to follow best storage practices more consistently, in contrast to large animal or mixed practices. This could be due to differences in clinical requirements and the physical set up of surgeries, which may warrant specific approaches to drug storage.^22^ For instance, small consultation room fridges in companion animal practices are generally separated from the main medical refrigerator, which allows for quick use and storage, thus better practice with keeping refrigerated drugs at the correct temperature.^23^ Further, as both veterinarians and veterinary nurses are generally present in small animal practices, drug storage practices are likely to be more thoroughly monitored and established in the surgery, which again may contribute to more robust practices in clinic.^24^ In contrast, in large animal practice, implementation of such measures is more complex. This is because the majority of consultations require on‐farm or home consultation visits,^22^ with veterinary surgeons often covering different geographical locations, requiring therefore vehicle storage of medicines, a practice that is discouraged because it is known to be susceptible to temperature changes.^25^, ^26^ Accordingly, in our study, many of the large animal practices reported using vehicle storage with cooling boxes, which exemplifies the potential for poorer storage practices. Yet, in our study most respondents were small animal practitioners and thus the sample size of large animal practices was small. Future studies with larger sample sizes, including more large animal practices, across the UK may help verify the extent to which this trend captures the reality of widespread veterinary drug storage practices.

Older professionals, particularly aged between 40–49 and 50+ years, scored statistically significantly higher for always following best drug storage practices, when compared to younger professionals. It is possible that this could be due to greater clinical experience and, thus, better performance on drug safety measures, which is consistent with our findings on clinical experience. However, as pointed out previously, older professionals are more likely to undertake managerial roles due to their clinical experience and have the financial means to become surgery owners. Therefore, the extent to which this finding in older professionals is mostly influenced by clinical experience or professional role within the clinic remains unknown. Future study is necessary to examine the effects of how different socio‐demographic factors, such as age and clinical experience, determine the individual role of potential risk factors for best drug storage practice in veterinary clinics.

There were no differences in response to storage practices among gender of the participants. Assessing gender differences in perception of job performance is essential to better understand the issue of gender inequality reported in the veterinary profession, particularly in senior management roles,^14^, ^27^ to ensure inclusive practices are maximised in veterinary practice. This is of increasing importance in the veterinary profession given that it has become highly femininised,^27^, ^28^ and the potential influential role senior management is likely to have in a clinic, influencing uptake of best practices. Therefore, further study is necessary to determine how different practices in clinic, including drug storage practices, are influenced by gender and how professionals can work towards narrowing gender inequality in professional practice; in particular, exploring which motivational factors are likely to play a crucial role in determining clinical performance. This could also help determine the extent to which gender could be a potential risk factor and how gender differences in drug storage practices could be addressed.

The RCVS regards a dispenser nurse as an essential role for effective drug storage practices in veterinary practice.^29^ Yet, as suggested by our results, the need for such a role in the veterinary industry appeared to have no significant impact on the abilities of professionals, with many practices not having a dispenser nurse on site. It is possible that this could have been due to a smaller sample size, thus limiting the number of practices that had a dispenser nurse. Indeed, less than 20% of surgeries had a dispenser nurse in our study. Another explanation could be the lack of prioritisation of this role in clinical practice, as often all clinical staff of a surgery need to multitask and carry out drug safety checks alongside clinical consultations and veterinary treatment. Yet, collective practice could elicit confusion, as staff may heavily rely on each other for checks and these may not necessary always occur. This could, for example, result in expiry dates of medications being missed or drugs being left out of the refrigerator and exceed manufacturers’ guidance. Therefore, while collective efforts reflect good clinical practice and shared responsibility, it is important that a designated member of staff within the surgery takes ultimate responsibility for safe drug storage practices. This enables cross‐checking of all drug safety practices within clinic, thus minimising the potential for confusion or malpractices in the surgery.

This study had several limitations. The study relied on professionals’ self‐reporting of practices, which may be subject to social desirability bias.^30^ Therefore, it is possible that participants’ responses may not necessarily reflect their professional performance, but their perceptions of best practice. So, participants may have been less willing to report deficiencies in drug storage practices due to peer pressure and instead reported what they believed would be best professional practice. This is well‐established in the human medical profession, where clinicians may under‐report errors in practice due to experiencing associated feelings of shame, guilt and embarrassment.^31^

While demographically the sample was considered likely to be representative of veterinary related professions, with a predominance of female professionals, the sample size was still limited. Particularly, greater recruitment of male participants could have proven valuable to determine the true extent of gender differences, as well as more veterinary nurses would give a more accurate picture of potential differences in professional practice. Further studies addressing professionals’ reasoning for responses could help provide further insight into how professional's attitudes and performance marry up in veterinary practice. In particular, studies that assess participants’ level of motivation towards their profession may help determine whether this is an important factor in general job performance and drug storage practices. Moreover, this study only took into account the opinions of veterinary professionals on drug storage practices. However, it is well‐established that in veterinary practices, non‐veterinary professionals such as pharmacists are also likely to work in dispensaries, especially outside the UK. Therefore, future research studies should also take into account the opinions of these professionals, as this is likely to provide a more comprehensive picture of industry practices.

While veterinary surgeries mostly seemed compliant with regulatory requirements on drug storage, practitioners should consider a more comprehensive approach to maximise the uptake of different measures collectively, in contrast to favouring certain measures, such as storage temperature over others. Likewise, socio‐demographic factors, particularly clinical experience and practice type, should be considered as potential risk factors by practices in order to maximise best practice. Further, the relative importance of having a designated veterinary dispenser nurse remains unknown in clinical practice; our study suggests that collective efforts in drug storage should be overseen by a member of clinical staff to enable cross examination of staff practices.

## AUTHOR CONTRIBUTIONS

This study was designed by Jordan Perry and Kelly Gouveia. Jordan Perry was responsible for data collection. Jordan Perry and Kelly Gouveia carried out data analysis, and the manuscript was conjointly written by both authors.

## CONFLICTS OF INTEREST

The authors declare they have no conflicts of interest.

## FUNDING INFORMATION

The authors received no specific funding for this work.

## ETHICS STATEMENT

The authors confirm that the ethical policies of the journal, as noted on the journal's author guidelines page, have been adhered to. This study was approved by the Department of Biological Sciences at the University of Chester research ethics committee.

## Supporting information



Supporting Information

## Data Availability

The data that support the findings of this study are not available publicly due to ethical and confidentiality constraints, and they can be made available on request from the corresponding author.
