# Supplementary material for: Assessing risk factors for drug storage practices in veterinary surgeries: A questionnaire study of UK veterinary professionals
Source: Vet Rec Open. 2025 Jan 31;12(1):e270005. doi: 10.1002/vro2.70005 (PMC11783155; doi:10.1002/vro2.70005)
Supplement: Supplementary file 1 — Supporting Information [file VRO2-12-e270005-s001.pdf]

Supporting Information
S1 Participation information sheet
Project title: Assessing risk factors for drug efficiency in current veterinary clinical practice

You are being invited to take part in a research study, provided by the university of Chester biological sciences department. Before you decide, it is important for you to understand why the research is being done and what it will involve. Please take time to read the following information carefully and discuss it with others if you wish. Please get in touch with us via the following email 1808770@chester.ac.uk, if you have any questions regarding this survey.

What is the purpose of the study?

To assess current storage practices of veterinary medicines in daily clinical practice to determine potential risk factors associated with socio-demographic characteristics of participants and their daily drug practices that could affect drug efficiency in veterinary practice.

Why have I been chosen?

You have been chosen because you fall into the role of either a Veterinary surgeon or Veterinary nurse who is practising within a veterinary surgery.

Do I have to take part?

It is up to you to decide whether or not to take part. If you decide to take part, you are still free to withdraw at any time and without giving a reason. A decision to withdraw at any time, or a decision not to take part, will not be detrimental to you in any way.

What will happen to me if I take part?

If you decide to take part, you will be given this information sheet to read and to sign as a participant in the study. Your participation in this research will provide insight into identifying potential risk factors for appropriate storage and use of veterinary medicines in the veterinary surgery.

What are the possible disadvantages and risks of taking part?

There are no disadvantages or any identifiable risks in undertaking this survey.

What are the possible benefits of taking part?

By taking part, you will be contributing to advancing knowledge and research in this area of scientific research.

What if something goes wrong?

If you wish to complain or have any concerns about any aspect of the way you have been approached or treated during the course of this study, please contact Professor Andrew Lawrence, Head of Department Biological Sciences, University of Chester, Parkgate Road, Chester, CH1 4BJ, 01244 513059.

Will my taking part in the study be kept confidential?

All information which is collected about you during the course of the research will be kept strictly confidential so that only the researcher carrying out the research and his/her supervisor will have access to such information. The results will be written up into a report as part of the researcher's BSc. Individuals who participate will remain anonymous in any subsequent report or publication.

Who may I contact for further information?

If you would like more information about the research before you decide whether or not you would be willing to take part, please contact by email 1808770@chester.ac.uk

Thank you for your interest in this research.

Socio-demographic characteristics of participants

1. What gender do you identify as?
   - Male
   - Female
   - Other

2. Are you a practising Veterinarian or Veterinary nurse?
   - Veterinarian
   - Veterinarian Nurse

3. What is your age group?
   - 20-29
   - 30-39
   - 40-49
   - 50+

4. What type of clinical practice do you work in?
   - Exotic (i.e. zoo, practice, sanctuary)
   - Large animal (including equine)
   - Small animal
   - Mixed

5. How many years have you spent working in clinical practice?
   - Less than 5
   - 5-9
   - 10-19
   - 20+

6. Does the practice have a designated dispensing nurse or veterinarian?
   1-Yes
   2-no

Please state the option that most accurately describes your daily practice storing Veterinary medicines in clinical practice.

7. All medications returned to the correct storage immediately after use
   1. Never
   2. Rarely
   3. Sometimes
   4. Often
   5. Always

8. Advised storage requirements (e.g. cool temperature – 8 to 15℃) for all medications are adhered to

    1. Never
    2. Rarely
    3. Sometimes
    4. Often
    5. Always

9. Is drug storage checked for current maximum and minimum temperatures?
    1. Never
    2. Rarely
    3. Sometimes
    4. Often
    5. Always

10. Medications are replaced when the manufacturers storage requirements are exceeded
    1. Never
    2. Rarely
    3. Sometimes
    4. Often
    5. Always

11. Do you record expenditure wastage in case a veterinary medicine is spoiled?

    1-Never
    2-Rarely
    3-Sometimes
    4-Often
    5-Always

12. When stock arrives is it checked for expiry date?

    1-Never
    2-Rarely
    3-Sometimes
    4-Often
    5-Always

13. Is there a system in place to record drug deliveries, expiration dates and usage within practice (i.e. computer database, paper documentation)?
    1. Never
    2. Rarely
    3. Sometimes
    4. Often
    5. Always

14. Are drugs stored within a vehicle overnight or for a period of time?
    1. Never
    2. Rarely
    3. Sometimes
    4. Often
    5. Always

Table S1 Sociodemographic characteristics of participants

**Socio-demographic questions (independent variable)**	**Number**	**Percentage**
What gender do you identify as?	70<br>109	Male 39.1<br>Female 61.0
Are you a practising Veterinarian or a Veterinary Nurse?	138<br>39	Veterinarian 78<br>Veterinary nurse 22
What is your age group?	41<br>48<br>45<br>45	20-29 years 22.9<br>30-39 years 26.8<br>40-49 years 25.1<br>50 + years 25.1
What type of clinical practice do you work in?	1<br>33<br>121<br>24	Exotic 0.6<br>Large animal 18.4<br>Small animal 67.6<br>Mixed 13.4
How many years have you spent working in clinical practice?	34<br>32<br>41<br>71	Less than 5 years 19.0<br>5-9 years 17.9<br>10-19 years 22.9<br>20+ years 39.7
Does the practice have a designated dispensing nurse or veterinarian?	33<br>148	Yes 18.2<br>No 81.8

Table S2 Associations between having a dispenser nurse and drug storage practices

				**Dispenser nurse**									
		**Yes**						**No**					
**Storage practices**	**Total n (%)**	**Never**	**Rarely**	**Sometimes**	**Often**	**Always**	**Total n (%)**	**Never**	**Rarely**	**Sometimes**	**Often**	**Always**	**P value**
Storage after use	33 (18.4)	0 (0)	1 (0.6)	0 (0)	12 (6.7)	20 (11.2)	146 (81.6)	0 (0)	3 (1.7)	8 (4.5)	61 (34.1)	74 (41.3)	P = 0.454
Temperature storage	33 (18.3)	0 (0)	0 (0)	0 (0)	7 (3.9)	26 (14.4)	147 (81.7)	0 (0)	1 (0.6)	2 (1.1)	24 (13.3)	120 (66.7)	P = 0.783
Temperature check	33 (18.3)	0 (0)	0 (0)	1 (0.6)	7 (3.9)	25 (13.9)	147 (81.7)	1 (0.6)	2 (1.1)	11 (6.1)	28 (15.6)	105 (58.3)	P = 0.807
Replaced medicines	33 (18.3)	0 (0)	2 (1.1)	4 (2.2)	10 (5.6)	17 (9.4)	147 (81.7)	1 (0.6)	3 (1.7)	20 (11.1)	42 (23.3)	81 (45.0)	P = 0.749
Recording expenditure wastage	32 (18.3)	2 (1.1)	4 (2.3)	5 (2.9)	7 (4.0)	14 (8.0)	143 (81.7)	14 (8.0)	15 (8.6)	26 (14.9)	32 (18.3)	56 (32.0)	P = 0.952
Expiry date check	33 (18.4)	0 (0)	0 (0)	3 (1.7)	9 (5.0)	21 (11.7)	146 (81.6)	0 (0)	8 (4.5)	13 (7.3)	33 (18.4)	92 (51.4)	P = 0.560
Recording expiry date	33 (18.4)	1 (0.6)	0 (0)	2 (1.1)	5 (2.8)	25 (14.0)	146 (81.6)	10 (5.6)	2 (1.1)	17 (9.5)	19 (10.6)	98 (54.7)	P = 0.687
Vehicle storage	33 (18.3)	20 (11.1)	2 (1.1)	3 (1.7)	3 (1.7)	5 (2.8)	147 (81.7)	97 (53.9)	3 (1.7)	6 (3.3)	21 (11.7)	20 (11.1)	P = 0.458

There was no statistical association between any of the storage practices and having a dispenser nurse at the practice.
